# *Trichoderma* spp. mediated induction of systemic defense response in brinjal against *Sclerotinia sclerotiorum*

**DOI:** 10.1016/j.crmicr.2021.100051

**Published:** 2021-07-24

**Authors:** Satyendra Pratap Singh, Chetan Keswani, Surya Pratap Singh, Estibaliz Sansinenea, Trinh Xuan Hoat

**Affiliations:** a Department of Mycology and Pathology, Institute of Agricultural Sciences, Banaras Hindu University, Varanasi 221005, India; b Department of Biochemistry, Institute of Science, Banaras Hindu University, Varanasi 221005, India; c Facultad De Ciencias Químicas, Benemérita Universidad Autónoma De Puebla, 72590 Puebla, Pue, México; d Plant Protection Research Institute, Duc Thang, Bac Tu Liem, Ha Noi, Vietnam

**Keywords:** Microbial Consortium, *Trichoderma*, *Sclerotinia sclerotiorum*, Phenolics, Brinjal

## Abstract

Induction of resistance to pathogen is associated with the colonization of root by *Trichoderma* spp. has been attributed as one of the major mechanisms contributing to pathogenic invasion. The present study sheds light on the defense network of brinjal plant bioprimed with *Trichoderma* spp. challenged with *Sclerotinia sclerotiorum*. Plants treated with dual inoculation of *Trichoderma harzianum* and *Trichoderma asperellum* triggered further synthesis of TPC under *S. sclerotiorum* challenge with maximum increment recorded at 72 hours. In consortium treated and pathogen challenged plants, a higher amount of shikimic acid was observed at 72 hours, whereas other phenolics showed little differences among the treatments. The consortium treatment showed significantly higher defense related enzymes (Phenylalanine Ammonia Lyase, Peroxidase and Polyphenol Oxidase) activity than other treatments. The study signifies how *Trichoderma* spp. reprograms the host's defense network to provide robust protection against *S. sclerotiorum*. In the present case, overall protection was provided to the brinjal plants against the attack of *S. sclerotiorum*.

## 1 Introduction

Brinjal is a common horticultural crop cultivated globally. It has good nutritional quality value. The crop is susceptible to many plant pathogens but a polyphagous soilborne plant pathogen S. sclerotiorum is one of the major pathogens causing severe crop loss especially during winter season. It has the potential of carpogenic germination and can infect the host at all stages of growth i.e. from seedling to its maturity. Tender twigs, flowers and fruits are the most susceptible to ascospore infection. The wide host range, and ability to produce sclerotia, which can persist in the soil for many years, greatly contributes in the development of the disease. Since the pathogen is soilborne in nature and sclerotia can persist for many years, soil treatment is one of the best methods to manage the disease, but soil treatment of large areas with chemical pesticides is very costly and may lead to many severe consequences for the human, animal and soil health. Biological control is the one of the most feasible, eco-friendly solutions for the management of this pathogen. The biocontrol agent, *Trichoderma* spp. is a genus of fungi characterized as opportunistic plant symbionts which colonize the plant roots. *Trichoderma* spp. directly interact with the pathogens as well as trigger gene expression in the host during biochemical cross talk between *Trichoderma* spp. and plant, which directly modulates plant metabolism, enabling plants to defend against the invading pathogens. During interaction of *Trichoderma*-plant-pathogen, biochemical changes occur in the plants such as deposition of lignin, increase in total phenolic content, changes in enzymes profiles like chitinase, β-1, 3-glucanase, peroxidase, phenyl alanine ammonia lyase and changes in phenylpropanoids in response to pathogen attack (Bisen et al., 2019; Jain et al., 2012; Keswani, 2015; Keswani et al., 2019; Keswani, 2016; Singh et al., 2013; Singh et al., 2014; Singh et al., 2017). It has been observed that induction of resistance to pathogen is associated with the colonization of roots by *Trichoderma* spp. (Harman et al., 2004; Keswani, 2016; Nguyen et al., 2019; Ram et al., 2019; Shoresh et al., 2005; Singh et al., 2011; Yedidia et al., 2003). The use of a consortium of two or more microbes may provide a better response over single microorganism formulation which may enhance the level and consistency of managing the disease by providing multiple mechanisms and may be more stable over wide range of environmental conditions (Abeysinghe, 2009; Basco et al., 2017; Fraceto et al., 2018; Keswani et al., 2019; Singh et al., 2013; Srivastava et al., 2010). Therefore, the main objective of this study was to evaluate the effect of *Trichoderma* spp. on the defense network of brinjal plants during *S. sclerotiorum* infection.

## 2 Materials and methods

### 2.1 Microbial cultures

In this study the phytopathogen *S. sclerotiorum* was isolated from the infected brinjal plant from the agricultural farm of Banaras Hindu University (Varanasi) and two biocontrol isolates *T. harzianum* (GenBank accession no. JN618343) and *T. asperellum* (GenBank accession no. JN618346) were used for seed biopriming. *T. harzianum* and *T. asperellum* were grown on Potato Dextrose Agar (PDA) dishes and incubated for 6 days at 27 ± 1 °C. The spores were harvested and the final concentration was adjusted to 2 × 10^8^ CFU/ml by sterile distilled water Analytical grade chemicals and solvents for experiments were obtained from E. Merck, Mumbai, India.

### 2.2 Greenhouse experiment

Seeds of brinjal (*Solanum melongena*; variety- Sweekar-321, a susceptible variety) were procured from the local market and were sanitized for 30 s with sodium hypochlorite (1%), washed twice with sterile water, and dried under a sterile stream of air. The sterilized seeds were grown in pots which were filled with sterilized soil. Seedlings were uprooted and treated with *Trichoderma* spp. suspension (seedling dip method with suspension of 2 × 10^8^ CFU/ml) for 30 min and transplanted in sterile soil (farm yard manure (FYM), vermicompost and sand in the proportion of (1:1:2)) according to the treatments, untreated seedlings served as control. The pots were kept in glasshouse under controlled conditions. The seedlings were challenged with pathogen one week after transplantation. The experiments were conducted with five treatments (T) and each treatment replicated thrice. T-1: Healthy control, T-2: *S. sclerotiorum* challenged control, T-3: *T. harzianum* + *T. asperellum* + *S. sclerotiorum*, T-4: *T. harzianum* + *S. sclerotiorum* and T-5: *T. asperellum* + *S. sclerotiorum*. Leaf samples were collected at every 24 h after pathogen inoculation (hapi) till 96 h. For each treatment, five pots containing single seedling was maintained. The experiment was designed in a completely randomized manner.

### 2.3 Total phenolic content (TPC)

Total phenol was estimated by the method described by Ragazzi and Veronese (1973). Sample preparation was done by homogenizing 1 g fresh leaf in 50% methanol left for 1 hour and then supernatant were collected after centrifugation. 100 µl of supernatant were taken and the volume was adjusted to 1.0 ml by adding distilled water; then 0.5 ml of Folin-Ciocalteau's phenol reagent (1 N) were added and mixed well. Thereafter 1.0 ml of Na_2_CO_3_ (20%) was added and vortexed. The reaction mixtures were left for 15 min at room temperature. Distilled water (10 ml) was added to the reaction mixture and it was vortexed. Absorbance was recorded at 725 nm and the results were expressed in mg gallic acid equivalent (GAE) g^−1^ fresh weight (FW).

### 2.4 HPLC analysis of phenolics

For HPLC analysis, sample preparation was done by using 1 g of fresh leaf samples harvested 0 h, 24 h, 48 h, and 72 h after inoculation of the pathogen were homogenized in 50% methanol (10 ml) and centrifuged for 15 min at 13,000 rpm. The supernatants were obtained by centrifugation and the phenolic content was separated by extraction with ethyl acetate. Residues were obtained by removing solvent dissolved in HPLC grade methanol for analysis of specific phenolics (254 nm). Separation was achieved by the method described by Singh et al., (2009). The solvent flow rate was 1.0 mL min^−1^. Separation of the compounds was achieved with water/acetonitrile (1:1 v/v) containing 1% glacial acetic acid in a gradient program, starting with 18% acetonitrile, changing to 32% at 10 min and finally to 50% at 20 min. For analysis HPLC system Shimadzu model LC-10A (Japan) was used and data were analyzed using Shimadzu Class VP series software by comparing the peak areas (max. 254 nm) of the samples with those of standard. Results are presented in the units of μg g^−1^ FW.

### 2.5 Assessment of defense-related enzymes

#### 2.5.1 Phenylalanine Ammonia Lyase (PAL)

For the analysis of PAL 0.5 g of leaf sample was homogenized in ice cold sodium borate buffer (0.1 M, pH 7.0) incorporated with 1.4 mM β-mercaptoethanol. Homogenized samples were centrifuged, and the supernatant was used as enzyme source for the study. Supernatant (0.2 ml) was mixed with 0.5 ml borate buffer (0.2 M, pH 8.7), distilled water (1.3 ml) and L-phenylalanine (0.5 ml, 0.1 M) and incubated for 30 min at 32 °C. Trichloroacetic acid (0.5 ml, 1 M) was added to finish the reaction. The absorbance was recorded at 290 nm as described by Brueske (Brueske, 1980) and the results were expressed as amount of formed trans-Cinnamic acid (µM t-CA mg^−1^ FW).

#### 2.5.2 Peroxidase (PO) assay

Peroxidase assay was performed following the method described by Hammerschmidt et al., (1982). In brief, 0.1 M phosphate buffer (5.0 ml, pH 7.0) was used for homogenization of leaf samples (0.5 g) and resulting supernatant was used for the enzyme assay. The reaction was started by adding 1.5 ml 0.05 M pyrogallol, enzyme extract (0.5 ml) and 1% H_2_O_2_ (0.5 ml). The results were noted as changes in absorbance (O.D.) at 420 nm and articulated as changes in the O.D. min^−1^ g^−1^ FW.

#### 2.5.3 Polyphenol Oxidase (PPO)

Analysis of PPO activity was done following the method described by Gauillard et al. (1993) in which homogenization of the leaf samples (0.5 g) was done in 5 ml of sodium phosphate buffer (0.1 M, pH 6.5). After centrifugation, the resulting supernatant was used as an enzyme source. The reaction was started by adding 0.4 ml catechol (0.01 M), 3.0 ml of sodium phosphate buffer (0.1 M, pH 6.5) and 0.4 ml of enzyme extract. The results were noted as changes in absorbance at 495 nm and articulated as changes in the O.D. min^−1^ g^−1^ FW.

### 2.6 Statistical analysis

Values from different experiments shown in table and figures were the average of at least three determinations. The data are expressed as mean of three replications ± standard deviations. The treatment averages were compared by DMRT with level of significance *p* ≤ 0.05, using SPSS version 20.

## 3 Results

### 3.1 Effect of *Trichoderma* spp. on the Total Phenolic Content (TPC)

**Fig. 1:**
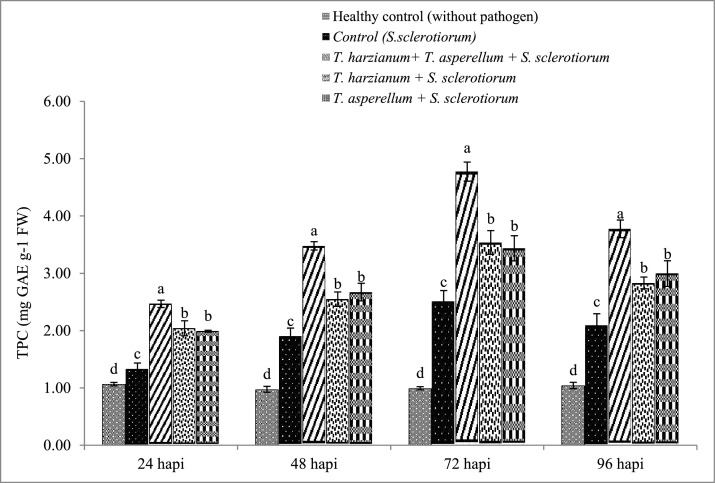
Changes in total phenol content in brinjal leaves after treatments with *T. harzianum* and *T. asperellum* singly as well as in combination and challenged with *Sclerotinia sclerotiorum*. Results are exhibited as means of three replicates and vertical bars shown in the figure represent standard deviations of the means. Significant differences among treatments depicted by different letters according to duncan's multiple range test at *p* ≤0.05.

The TPC content was evaluated in control and in plants treated with *Trichoderma* isolates and challenged with *S. sclerotiorum* at regular intervals from 24 h to 96 h after pathogen inoculation. Increasing trends were recorded in *Trichoderma* spp. treated plants and *S. sclerotiorum* pathogen challenged plants up to 72 h. All the plants treated with *Trichoderma* spp. showed significantly higher TPC than the control. The maximum TPC was recorded at 72 h in the *T. harzianum* + *T. asperellum* treated and *S. sclerotiorum* pathogen challenged plant (4.77 mg GAE g^−1^ FW) followed by individual *T. harzianum* (3.54 mg GAE g^−1^ FW) and *T. asperellum* (3.44 mg GAEg^−1^ FW) treated and pathogen challenged plant while in *S. sclerotiorum* pathogen inoculated control it was 2.51 mg GAE g^−1^ FW (Fig. 1). As shown in Fig. 1, it is clear that the dual inoculation of *T. harzianum* and *T. asperellum* triggered higher synthesis of TPC than single isolate application.

### 3.2 Effect of *Trichoderma* isolates on free phenolic profile

The quantitative analysis of free phenolic acid content was done through HPLC, as shown in Table 1A–C and Fig. 3A-C. From the results it is clear that there were quantitative differences among the treatments. In this analysis, levels of phenolics such as shikimic acid, gallic acid, trans-Chlorogenic acid, tannic acid, syringic acid, rutin, p-Coumaric acid, 3,4-Dihydroxycinnamic acid, ferulic acid, quercetin and kaempferol were recorded. The results indicated that shikimic acid was present in the highest amount among all studied phenolics during the study period which ranges from 338.93 to 1709.39 µg g^−1^ FW. At 72 h in consortium treated and pathogen inoculated plants showed 2.82 times and 4.91 times higher shikimic acid amount than the untreated pathogen inoculated control (T-2) and healthy control (T-1) respectively, whereas gallic acid and t-Chlorogenic acid also found significantly higher amount than untreated pathogen inoculated. Single isolate application of *Trichoderma* treated plants (T-4 and T-5) also showed higher amount of shikimic acid, gallic acid and t-Chlorogenic acid at 72 h and 96 h. (Table 1B and 1C). The observations indicated that the consortium treated plants challenged with pathogen T-3 treatment (*T. harzianum* + *T. asperellum* + *S. sclerotiorum*) showed significantly higher shikimic acid amount at 48, 72 and 96 h (Table 1A, 1B and 1C) whereas treatment T-4 (*T. harzianum* + *S. sclerotiorum*) and T-5 (*T. asperellum* + *S. sclerotiorum*) showed significantly higher at 48 and 72 h (Table 1A and 1B). It has been observed that the amounts of other phenolics such as tannic acid, syringic acid, rutin, p-Coumaric acid, 3,4-Dihydroxycinnamic acid, ferulic acid, quercetin and kaempferol had little differences among the treatments at all time periods.

**Table 1A:** Status of phenolic acids in leaves of brinjal as influences by various treatments at time intervals A: 48 hapi, B: 72 hapi and C: 96 hapi.

Treatments/Phenolics	T1	T2	T3	T4	T5
Shikimic acid	338.93±42.94c	432.98±51.65bc	718.79±62.69a	524.42±52.59b	447.39±37.69b
Gallic acid	60.29±8.86a	32.04±8.93b	60.36±10.73a	43.67±9.51ab	34.25±4.70b
Trans-Chlorogenic acid	28.93±5.37b	19.72±3.47b	48.39±9.96a	17.65±2.81b	25.06±5.35b
Tannic acid	ND	18.40±3.49b	80.23±13.42a	20.88±4.44b	68.34±17.75a
Syringic acid	50.64±9.08bc	46.45±5.26c	95.08±17.89a	25.42±3.66d	68.82±8.17b
Rutin	35.99±4.47c	66.39±3.40b	113.34±18.78a	23.39±7.19c	97.72±17.97a
p-Coumaric acid	14.80±1.89a	13.05±0.90a	3.13±0.85b	15.52±3.48a	6.21±1.98b
3,4-Dihydroxycinnamic acid	ND	3.54±0.45b	6.52±1.00a	ND	3.68±0.26b
Ferulic acid	5.27±0.81a	3.14±0.89b	3.64±0.49b	3.71±0.72b	2.50±0.53b
Quercetin	ND	0.16±0.04bc	0.99±0.32a	0.39±0.09b	0.26±0.04bc
Kaempferol	0.86±0.17a	0.56±0.18a	0.81±0.27a	0.63±0.10a	0.60±0.12a

**Table 1B:** 

Treatments/Phenolics	T1	T2	T3	T4	T5
Shikimic acid	348.40±41.23d	605.25±87.91c	1709.39±123.98a	829.03±64.97b	885.32±76.42b
Gallic acid	39.82±8.45c	38.31±8.20c	108.30±17.33a	56.45±13.67bc	73.44±18.10b
Trans-Chlorogenic acid	17.65±4.54d	36.82±5.08c	74.59±9.30a	45.66±7.69bc	59.38±8.64b
Tannic acid	18.18±5.35c	121.30±17.76a	121.64±18.01a	63.03±8.96b	128.55±19.13a
Syringic acid	55.29±13.59c	84.93±13.88ab	92.77±5.53a	65.60±13.51bc	50.18±10.00c
Rutin	57.12±8.95c	122.93±17.47ab	129.62±17.62a	94.91±8.95b	122.45±14.54ab
p-Coumaric acid	4.90±0.85b	7.90±1.79a	3.93±0.63b	2.99±0.36b	8.75±1.87a
3,4-Dihydroxycinnamic acid	3.80±0.82a	4.87±0.81a	5.03±0.89a	4.03±0.88a	4.24±0.82a
Ferulic acid	3.38±0.26a	3.12±0.10a	2.21±0.18b	3.32±0.39a	3.41±0.36a
Quercetin	0.13±0.02b	0.43±0.10a	0.48±0.08a	0.43±0.08a	0.17±0.03b
Kaempferol	0.62±0.24b	0.82±0.09b	1.33±0.26ac	0.09±0.01c	0.52±0.09b

**Table 1C:** 

Treatments/Phenolics	T1	T2	T3	T4	T5
Shikimic acid	398.70±55.72c	570.91±63.53b	1031.12±71.56a	604.50±67.16b	576.75±71.55b
Gallic acid	32.18±5.49b	38.60±7.29b	94.67±18.00a	70.66±17.98a	45.55±5.20b
Trans-Chlorogenic acid	38.43±6.97ab	24.143.52c	43.45±6.98ab	44.06±3.60a	32.45±3.41bc
Tannic acid	72.52±8.68b	88.55±11.85b	102.44±19.04ab	127.98±20.56a	89.41±17.10b
Syringic acid	43.23±5.54b	72.51±9.04a	71.22±9.46a	75.35±10.55a	59.69±8.26ab
Rutin	96.91±17.89ab	90.18±8.99b	94.15±17.83ab	123.06±17.88a	78.89±9.07b
p-Coumaric acid	3.43±0.40c	7.47±1.06a	4.96±0.79b	8.37±0.49a	8.51±0.78a
3,4-Dihydroxycinnamic acid	4.78±0.88a	5.71±0.88a	2.31±0.28b	6.23±0.72a	6.17±0.88a
Ferulic acid	2.81±0.59b	1.92±0.61bc	1.49±0.25c	5.41±0.50a	4.54±0.36a
Quercetin	0.38±0.09b	4.96±0.53a	0.76±0.21b	0.75±0.18b	0.27±0.03b
Kaempferol	0.14±0.03c	1.75±0.41a	0.68±0.09b	1.92±0.26a	0.52±0.09bc

T-1 Healthy control, T-2 Pathogen (*Sclerotinia sclerotiorum*) inoculated control, T-3 *T. harzianum* + *T. asperellum* + *Sclerotinia sclerotiorum*, T-4 *T. harzianum* + *Sclerotinia sclerotiorum*, T-5 *T. asperellum* + *Sclerotinia sclerotiorum*. Different letters in the row data indicate significant difference between the phenolic compounds across the treatments according to DMRT at *p* ≤ 0.05.Data represents mean± standard deviation of three replicates. ND: Not detected

### 3.3 Effect of *Trichoderma* isolates on defense related enzymes

#### 3.3.1 Phenylalanine Ammonia Lyase

**Fig. 2:**
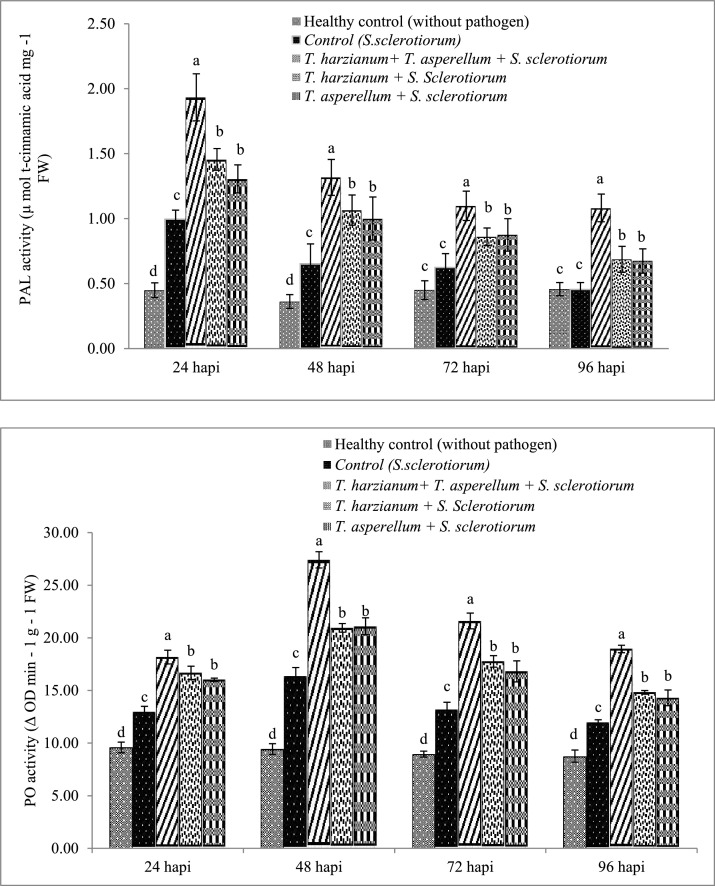

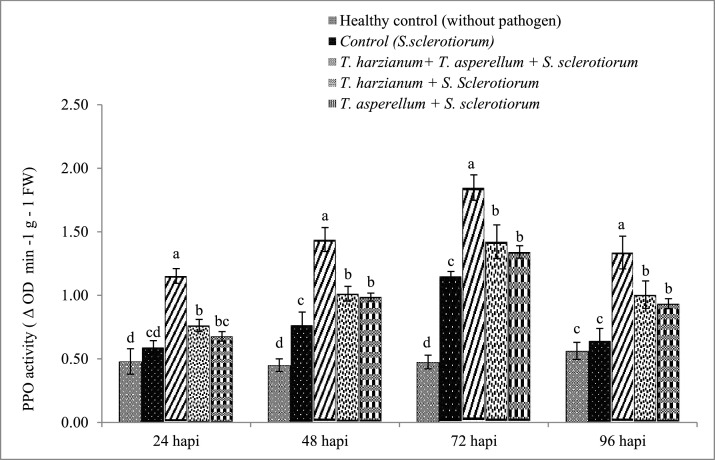
Changes in Phenylalanine Ammonia Lyase (A), Peroxidase (B) and Polyphenol Oxidase activities (C) in brinjal leaves after treatments with *T. harzianum* and *T. asperellum* singly as well as in combinations and challenged with *Sclerotinia sclerotiorum*. Results are exhibited as means of three replicates and vertical bars shown in the figure represents standard deviations of the means. Significant differences among treatments depicted by different letters according to Duncan's multiple range test at *p* ≤0.05.

Results indicated that the consortium of *T. harzianum* + *T. asperellum*, enthused the PAL activity after pathogen *S. sclerotiorum* inoculation. The highest PAL activity (1.93 μM t-Cinnamic acid mg^−1^ FW) was recorded at 24 h in the consortium treatment followed by single *T. harzianum* (1.46 μM t-Cinnamic acid mg^−1^ FW) and *T. asperellum* (1.31 μM t-Cinnamic acid mg^−1^ FW) treated plants, these were significantly higher than untreated pathogen inoculated treatment (1.00 μM t-Cinnamic acid mg^−1^ FW) (Fig. 2A). As shown in Fig. 2A it is clear that the consortium treatment showed significantly higher PAL activity than other treatments at all time periods i.e. 48, 72 and 96 h. A gradual decline was observed in the PAL activity after 24 h.

**Fig. 3:**
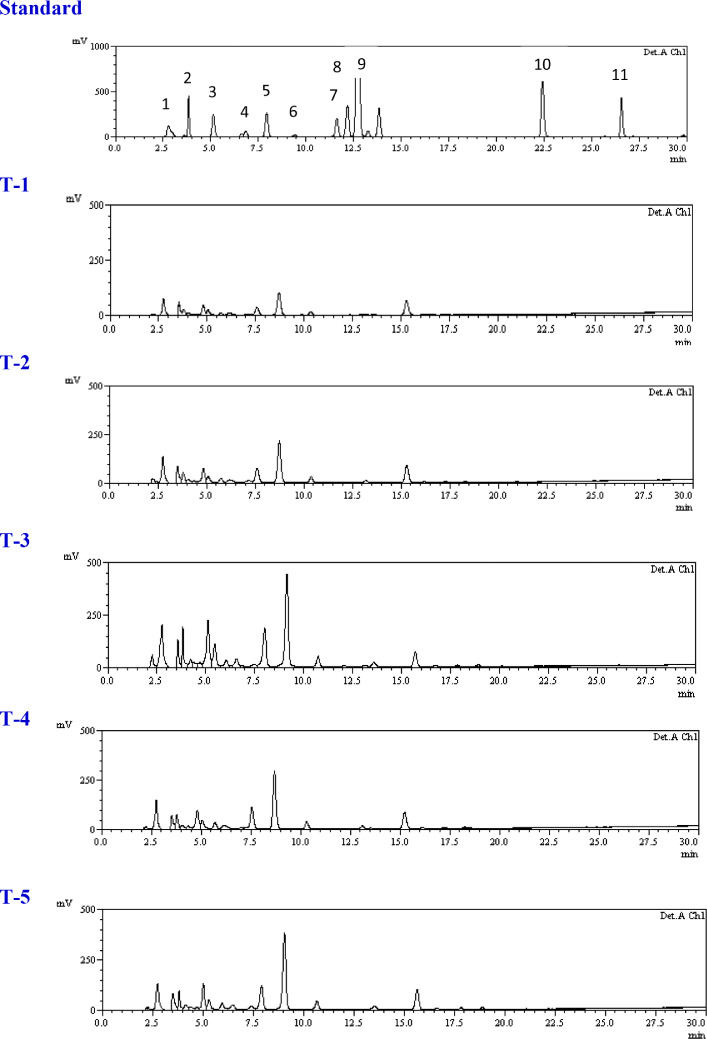

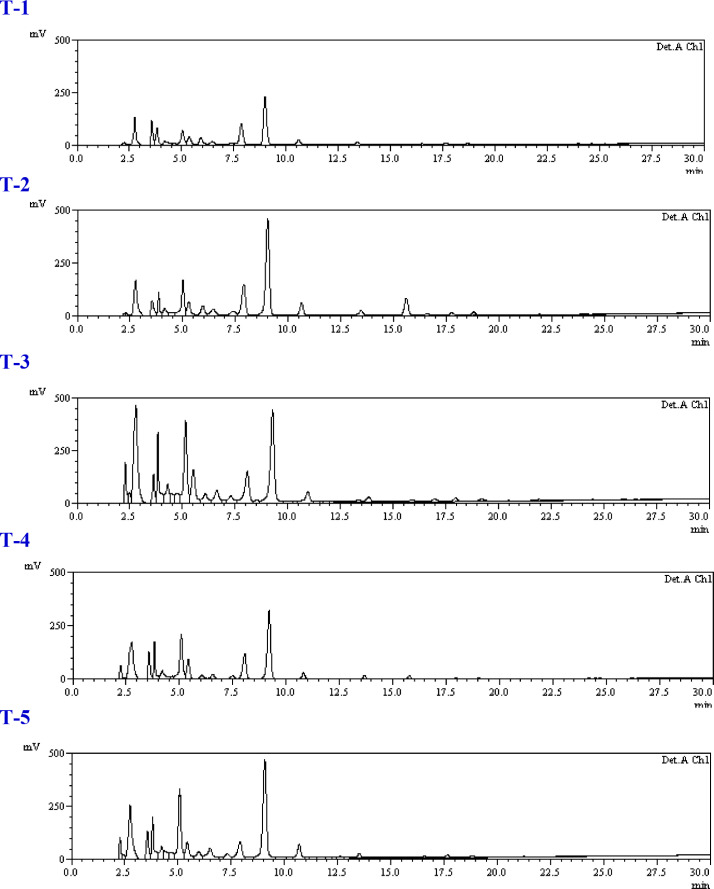

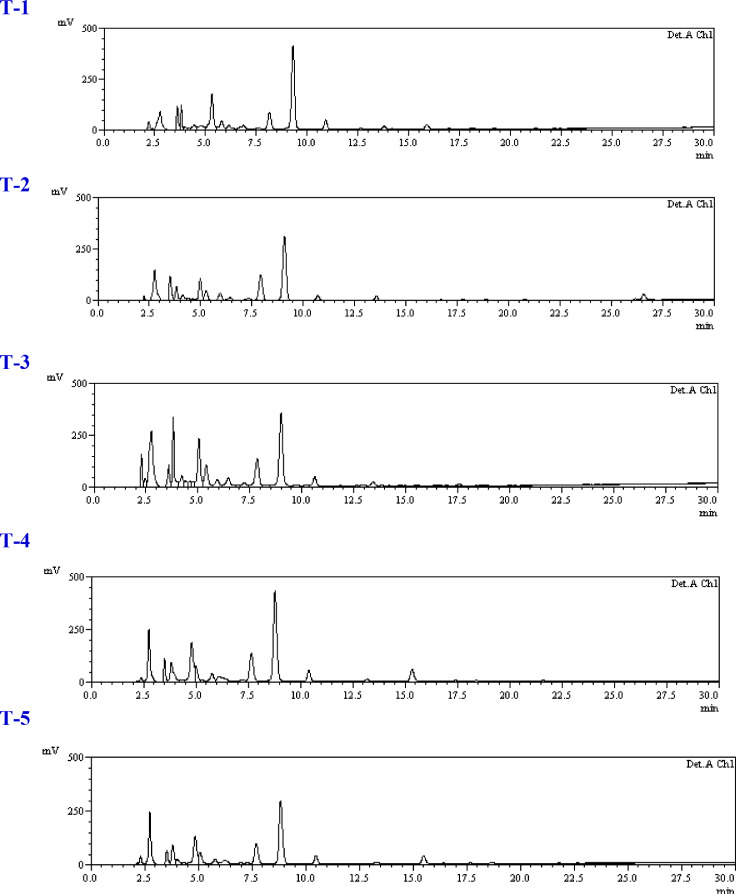
Phenolic profile across various treatments (T-1 to T-5) in brinjal leaves at particular time intervals i.e. A: 48 hapi, B: 72 hapi and C: 96 hapi. 1: shikimic acid, 2: gallic acid, 3: t-Chlorogenic acid, 4: tannic acid, 5: syringic acid, 6: rutin, 7: p-Coumaric acid, 8: 3,4-Dihydroxy.

#### 3.3.2 Peroxidase (PO) assay

Fig. 2B shows enhanced PO activity in the treatments with *Trichoderma* inoculated with pathogen. The activity was recorded up to 96 h. The PO activity increases from 24 h to 48 h. The maximum PO activity was recorded at 48 h in consortium (*T. harzianum* + *T. asperellum* + *S. sclerotiorum*) treated plants followed by single *T. asperellum* and *T. harzianum* treated plant which were 1.67, 1.29 and 1.28 times higher than the untreated pathogen inoculated control respectively. The results shown in Fig. 2B indicated that the activity of PO was significantly higher in *Trichoderma* treatments (a and b) than untreated pathogen inoculated control (c) and untreated uninoculated healthy control (d) (Fig. 2B).

#### 3.3.3 Polyphenol Oxidase (PPO)

The PPO activity was increased in consortium (*T. harzianum* + *T. asperellum* + *S. sclerotiorum*) treated plants. The maximum PPO activity was recorded 72 h in the consortium treated plants followed by single *T. harzianum* and *T. asperellum* treated plants which were 1.61, 1.23 and 1.16 times higher than pathogen inoculated control, respectively. An increasing trend was recorded from 24 h to 72 h. All the *Trichoderma* treated plants (a and b) exhibited higher PPO activity than pathogen inoculated control (c) and uninoculated healthy control (d) (Fig. 2C). From the data it is clear that *Trichoderma* spp. induce the accumulation of defense related enzymes in the treated plants.

## 4 Discussion

Irrational and excessive use of chemical pesticides and fertilizers has irreversibly contaminated the fertile soil and ground water posing a severe threat to human and environmental health. *Trichoderma* spp. are opportunistic, avirulent plant symbionts (Harman et al., 2004) which play a pivotal role in sustainable agriculture. Use of single species formulation of *Trichoderma* is a very common practice. The use of a consortium of *Trichoderma* spp. having different characteristics provides new avenues to explore its applications for agricultural purposes. In this study we use two *Trichoderma* isolates of different characteristics, individually and in consortium form and evaluate the defense related compounds in brinjal plants after pathogen inoculation. Results indicated that during the plant microbe interaction the biochemical activity in the plants was altered.

The results obtained in this study revealed that the consortium of *Trichoderma* showed better activity that induced defense related enzymes such as PAL, PO, PPO and different phenolics with greater amount of each of them than the amount of individual treatment of one of them. Phenolic compounds found in plants have antimicrobial properties and serve as signaling molecules (Hammerschmidt, 2005). Phenolics are a large and chemically diverse family of compounds from simple phenol to large and complex polymers such as tannins and lignin. In this study *Trichoderma* treated plants either individual or in consortium form, showed significantly higher amount of TPC than control. Recently, it has been reported that the use of consortium of *Trichoderma, Pseudomonas* and *Bacillus* in pea (Jain et al., 2012), *Trichoderma, Pseudomonas* and *Rhizobium* in chickpea (Singh et al., 2013) and *Trichoderma* spp. in tomato (Singh and Singh, 2015) activates the phenylpropanoid pathway during pathogen invasion. It has also been reported that chickpea seed treatment and soil amendment of *T. viride* increased the TPC in *Machrophomina phaseolina* challenged plants (Singh et al., 1998).

In this study, we targeted different phenolics such as shikimic acid, gallic acid, t-Chlorogenic acid, tannic acid, syringic acid, rutin, p-Coumaric acid, 3,4-Dihydroxycinnamic acid, ferulic acid, quercetin and kaempferol. Shikimic acid plays important role in the synthesis of gallic acid, phenylalanine, cinnamic acid, p-Coumaric acid, ferulic acid, syringic acid and phenylpropanoids and ultimately lignification in the plants (Singh and Singh, 2015). A key product of phenylpropanoid pathway involved in host resistance against pathogens is t-Cinnamic acid synthesized from shikimic acid via. L-phenylalanine (Karthikeyan et al., 2006; Mandal & Mitra, 2007). Salisbury and Ross (Salisbury; Ross, 1986) reported that gallic acid is biotransformed into potent antimicrobial gallotannins whereas, Singh et al. (2010) reported that phenolics like t-Chlorogenic acid, ferulic acid and protocatechuic acid have potent antifungal properties.

In the current study we found that shikimic acid, gallic acid, t-Chlorogenic acid, tannic acid, syringic acid and rutin were recorded much higher than other five phenolics (Table 1A, 1B, 1C). In the consortium (T-3) treated, single *Trichoderma* (T-4 and T-5) treated and pathogen inoculated treatment showed higher shikimic acid amount than untreated pathogen inoculated control (T-2). We prospect that the synthesis of relatively higher amount of shikimic acid in the *Trichoderma* treated plants especially in consortium after pathogen inoculation indicated the activation of induced systemic response in the host as shikimic acid pathway is directly involved in the synthesis of free phenols and acts as precursor for synthesis of tannins, flavonoids, and lignin. Similarly, induction in the production of gallic acid, t-Chlorogenic acid and syringic acid in *Trichoderma* treated and pathogen challenged treatment also supports the reducing disease caused by *S. sclerotiorum*. It has been reported that chlorogenic acid is the ester of phenolics and has antiviral, antibacterial and antifungal activities (Bowles & Miller, 1994; Clifford et al., 2003; De Sotillo et al., 1998; Jassim & Naji, 2003).The rapid enhancement in phenolics may be due to the rapid root colonization and biochemical cross talk between *Trichoderma* and brinjal plants during pathogen infection.

Harman et al., and Shoresh et al., reported that *Trichoderma* spp. produce signaling molecules that showed high sensitivity in several plants (Harman et al., 2004; Shoresh & Harman, 2008). The increase in phenolic content in plants is responsible for lignin deposition. Our results are in agreement with the findings in maize root, treated with *T. harzianum* strain T-22 (Bigirimana et al., 1997). Similar observations were also recorded by Singh et al. (2014) in which triple microbe treatment showed phenolics accumulation in chickpea.

In phenylpropanoid metabolism, phenylalanine ammonia lyase plays an important role in the synthesis of anti-microbial secondary metabolites in the plants. PAL is an important enzyme in the synthesis of important phenolics. The reaction pathway consists in the conversion of phenylalanine to t-Cinnamic acid catalyzed by PAL then, t-Cinnamic acid is converted into p-Coumaric acid after addition of hydroxyl group and after another addition p-Coumaric acid gives ferulic acid. These sequential steps of conversion of phenylalanine are the responsible for the formation of lignin, tannin, flavonoids and isoflavonoids, which are the plant's weapons working against pathogen invasion. Treatment of plant with bioagents and their metabolites stimulates the phenylpropanoid pathway for the synthesis of different chemicals helping to produce higher amount of phenolics which act as precursor molecules for lignin synthesis (De Ascensao & Dubery, 2000>; Singh et al., 1998). Gallou et al. (2009) reported an induction of PAL gene expression on potato plant treated with *T. harzianum* Rifai MUCL 29707, against *R. solani* during early hours of post-infection. In another study, it was observed that *Trichoderma* upregulated Pal1 gene which is responsible for encoding PAL (Shoresh & Harman, 2008; Shoresh et al., 2005). In this study, we also found an increase in the level of PAL in the *Trichoderma* treated plants which is in agreement with the various other reports (Harman et al., 2004; Karthikeyan et al., 2006; Singh and Singh, 2015; Yedidia et al., 1999). Significantly higher amount of PAL activity was recorded in all *Trichoderma* treated plants (T-3, T-4, T-5) than pathogen inoculated control (T-2), which is similar result to the works reported in pea, chickpea and tomato, respectively (Jain et al., 2012; Singh et al., 2013; Singh and Singh, 2015). Peroxidase (PO) is another important component which is also responsible for the lignin synthesis and to strengthen the plants against pathogen (Bruce; West, 1989). The increased PO activity often correlated with the oxidation of phenolic substance in infected and resistant plants. The direct involvement of PO in the defense reactions of plant is that PO inhibits the fungal growth (Macko et al., 1968). During pathogen infection, PO activity was elicited in different plants such as tomato (Nandakumar et al., 2001; Ramamoorthy et al., 2002). It has been observed that upon treatment with plant growth promoting rhizobacteria (PGPR), peroxidase was elicited in cucumber (Chen et al., 2000), while inoculation of *T. harzianum* in cucumber root induced peroxidase activity in leaves (Yedidia et al., 1999). In this study, we also recorded significantly higher PO activity in the leaves of brinjal plant treated with a consortium of Trichoderma and singly *Trichoderma* treated plant challenged with *S. sclerotiorum* than untreated pathogen inoculated control. Another enzyme PPO catalyzes the oxidation of phenolic substances. The presence of phenolics and their oxidation products are toxic to the pathogen and inhibits tits growth. In this study, we recorded maximum PPO activity *Trichodrema* treated plants. Consortium of *Trichoderma* (T-3) treated treatment showed significantly higher PPO activity than other (Chen et al., 2000), also reported the effect of several rhizobacteria and *Pythium aphanidermatum* on PPO activity in cucumber. Coconut plant treated with a mixture of *Pseudomonas fluorescens, T. viride* and chitin showed rapid increase in PO and PPO activity against Ganoderma (Karthikeyan et al., 2006). Other examples can be mentioned such as the use of triple consortium of *Trichoderma, Pseudomonas* and *Bacillus* in pea (Jain et al., 2012), *Trichoderma, Pseudomonas* and *Rhizobium* in chickpea (Singh et al., 2013) and consortium of *Trichoderma* spp. (Singh and Singh, 2015) which increase the level of TPC, PAL, PO and PPO activity during pathogen invasion.

From the recorded data of this study it can be concluded that the seedling dip before transplanting with consortium of *Trichoderma* was more beneficial than inoculation with single isolate. The *Trichoderma* consortium treated seedlings showed higher accumulation/synthesis of defense related compounds in the brinjal leaves than single *Trichoderma* when challenged with pathogen. The findings of this study can be used to exploit the beneficial effects of treatment with *Trichoderma* consortium in farmers’ field.

## Declaration of Competing Interest

The authors have declared no conflict of interest.

## Appendix Supplementary materials

Image, application 1
