# Supplementary material for: Trichoderma spp. mediated induction of systemic defense response in brinjal against Sclerotinia sclerotiorum
Source: Curr Res Microb Sci. 2021 Jul 24;2:100051. doi: 10.1016/j.crmicr.2021.100051 (PMC8610364; doi:10.1016/j.crmicr.2021.100051)
Supplement: Supplementary file 1 [file mmc1.docx]

**Supplementary Data**

**FIGURE 3A:**

**Standard**


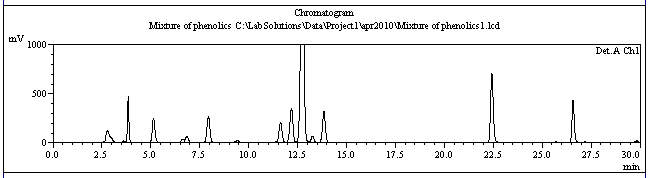


8

9

10

11

6

7

5

4

3

2

1


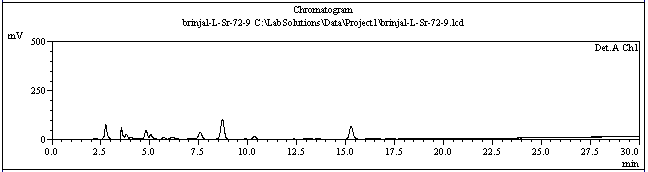
**T-1**


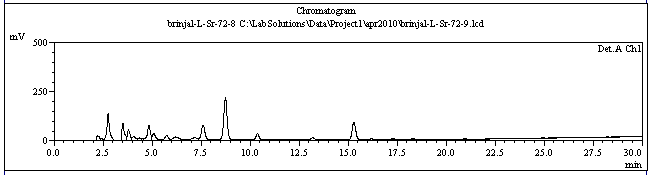
**T-2**

**T-3**
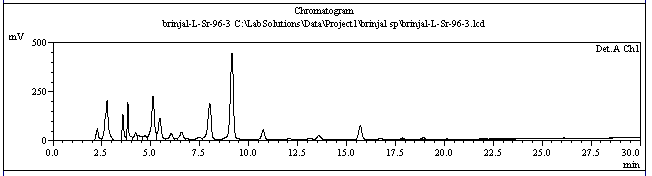


**T-4**
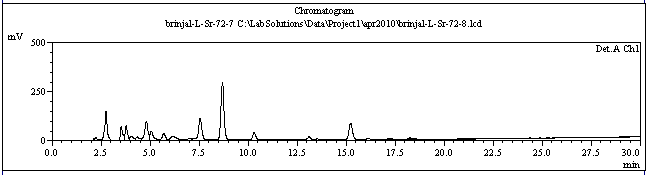


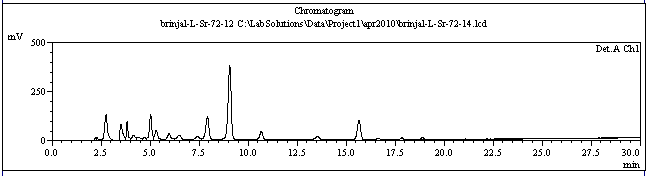
**T-5**

**FIGURE 3B**


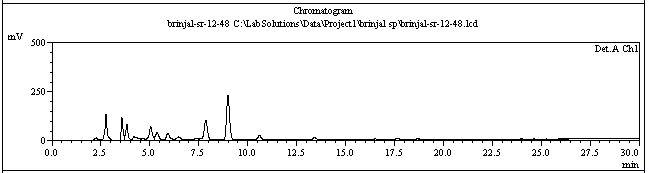
**T-1**


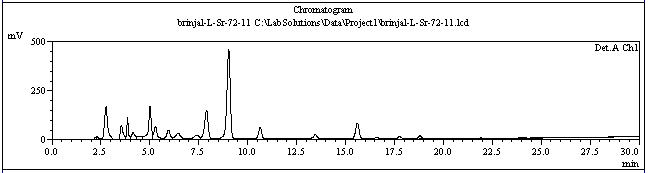
**T-2**


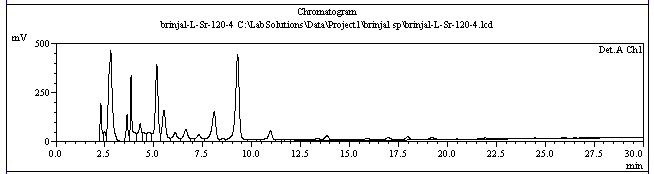
**T-3**


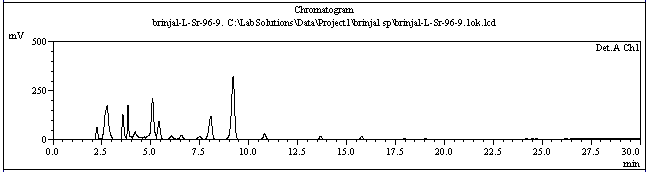
**T-4**


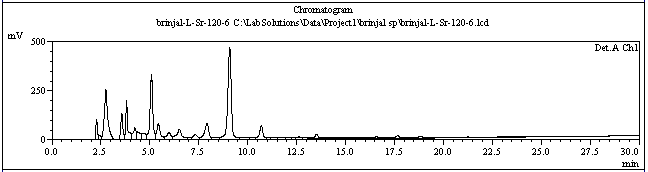
**T-5**

**FIGURE 3C**


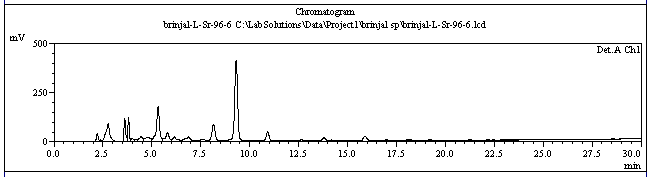
**T-1**


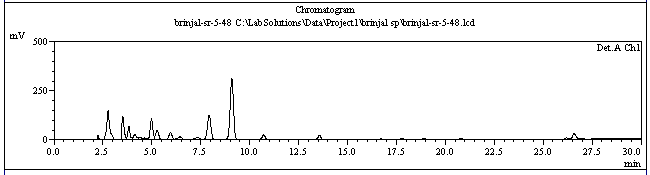
**T-2**


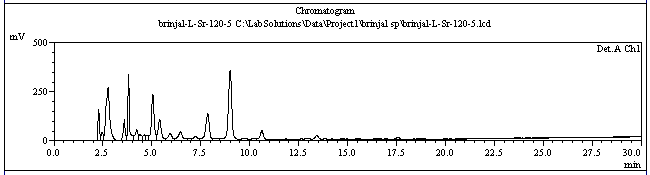
**T-3**


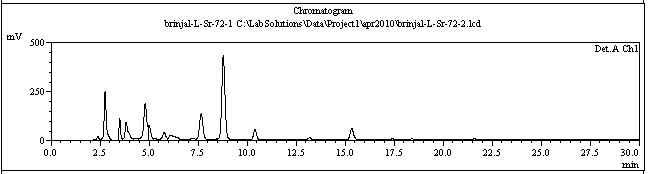
**T-4**


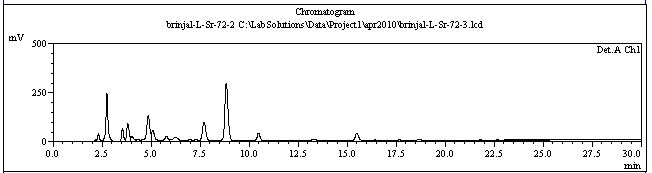
**T-5**

**Supplementary Data (Figure 4)**: Accumulation pattern of six major phenols (A) shikimic acid, (B) Gallic acid (C) *t*-Chlorogenic acid (D) Tannic acid (E) Syringic acid (F) Rutin in different microbial treatments (T-1-T-5) at 48 hapi, 72 hapi and 96 hapi in leaves of brinjal. Results are exhibited as means of three replicates and vertical bars shown in the figure represents standard deviations of the means. Significant differences among treatments depicted by different letters according to Duncan’s multiple range test at *P ≤0.05*.

**FIGURE 4A:**

**FIGURE 4B:**

**FIGURE 4C:**

**FIGURE 4D:**

**FIGURE 4E:**

**FIGURE 4F:**
